# From behavior to belonging: reframing exercise participation as a psychosocial pathway to active aging

**DOI:** 10.3389/fpubh.2025.1726308

**Published:** 2026-02-05

**Authors:** Soo-Jin Choi, Fang Zheng

**Affiliations:** Zhejiang University, Hangzhou, China

**Keywords:** active aging, eudaimonic wellbeing, exercise participation, psychosocial health, public health policy, social ecology, social connectedness

## Abstract

Traditional public health approaches often conceptualize physical exercise as a behavioral determinant of physical health. Yet, the psychosocial mechanisms through which exercise participation enhances wellbeing in later life remain insufficiently understood. This study reframes exercise participation as a *psychosocial ecosystem* that simultaneously fulfills eudaimonic (psychological) and relational (social) needs, promoting active aging through interconnected mental and social pathways. Drawing on eudaimonic wellbeing theory and the social ecology of aging, we tested a dual-mediation model linking exercise participation, psychological wellbeing, social connectedness, and life satisfaction among 412 older adults in South Korea. Structural equation modeling confirmed that exercise participation enhanced life satisfaction both directly and indirectly via psychological wellbeing (*β* = 0.20, *p* < 0.001) and social connectedness (*β* = 0.17, *p* < 0.001). These psychosocial mediators accounted for 43% of the total effect, underscoring the interdependence of emotional and social health in later life. The findings advance active-aging research by bridging behavioral health and social integration theories, positioning exercise as a *public health catalyst* for mental resilience and social cohesion. Public health strategies should therefore integrate psychological empowerment and social inclusion within community-based exercise initiatives to foster eudaimonic wellbeing and social vitality among aging populations.

## 1 Introduction

### 1.1 Background: population aging and active aging

As population aging accelerates globally, societies face growing pressure not only to extend life expectancy, but also to improve the quality of life in later years (1). In response, the World Health Organization has introduced the concept of active aging, defined as optimizing opportunities for health, participation, and security to enhance wellbeing as people grow older (2). Within this framework, physical activity has emerged as a key factor in maintaining both physical and mental health (3). Yet, despite its widely acknowledged value, much of the academic and policy discourse still approaches active aging primarily through a biomedical lens—emphasizing disease prevention and functional ability, often at the expense of more holistic considerations of well-being (4).

### 1.2 Limitations of existing research

A substantial body of research has confirmed that regular exercise yields benefits for both physical health and emotional wellbeing. However, much of this work has conceptualized physical activity primarily as a behavioral health strategy, focusing on outcomes such as improved muscle strength, reduced chronic disease risk, or lower depressive symptoms (5). These approaches often overlook the deeper psychological and social processes through which exercise contributes to meaning, belonging, and overall wellbeing in later life. Addressing this gap requires a shift from a predominantly biomedical focus to a more psychosocial perspective that recognizes exercise as an experience embedded in relational and emotional contexts.

### 1.3 Theoretical framework

To address this gap, the present study draws upon two theoretical perspectives. First, the theory of eudaimonic wellbeing emphasizes psychological dimensions such as autonomy, purpose, and personal growth as essential to human flourishing—beyond mere hedonic pleasure (6). In this view, exercise may serve as a platform for older adults to maintain a sense of purpose and self-development.

Second, the social ecology of aging considers wellbeing as a product of the fit between individuals and their social environments (7). From this perspective, group-based exercise activities or community fitness programs provide more than physical benefits—they create opportunities for meaningful social connection, emotional support, and belonging.

By integrating these perspectives, this study conceptualizes exercise participation as a psychosocial ecosystem—an environment where psychological and social dimensions of wellbeing are cultivated together (8).

### 1.4 Research objectives and hypotheses

Guided by this integrated framework, the present study sets out to:

1. Examine the associations between exercise participation, psychological wellbeing, social connectedness, and life satisfaction in older adults.
2. Test whether psychological wellbeing and social connectedness serve as mediators in the relationship between exercise and life satisfaction.
3. Explore how psychosocial factors can inform sustainable approaches to active aging.

Based on these aims, we propose the following hypotheses:

*H1*: Exercise participation positively influences psychological wellbeing.

*H2*: Exercise participation positively influences social connectedness.

*H3*: Psychological wellbeing positively influences life satisfaction.

*H4*: Social connectedness positively influences life satisfaction.

**Figure 1:**
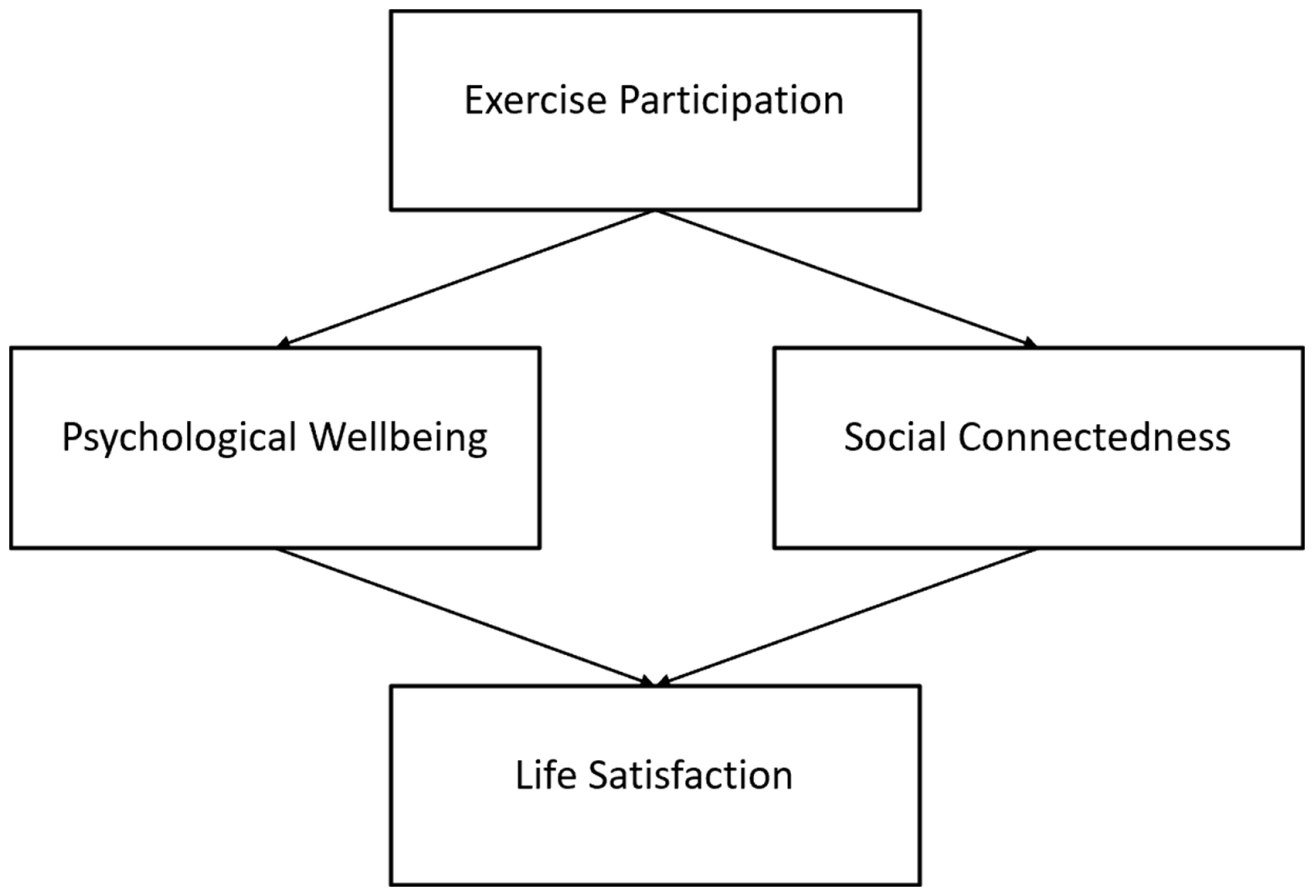
Conceptual model showing the dual psychosocial pathways from exercise participation to life satisfaction via psychological wellbeing and social connectedness.

Together, these hypotheses form a dual-mediation model, illustrating the psychological and social mechanisms through which exercise participation may promote life satisfaction. This reframing contributes to active aging research by integrating both individual and relational dimensions of wellbeing into a cohesive psychosocial framework. Figure 1 presents the conceptual framework of this study, outlining the hypothesized dual psychosocial pathways linking exercise participation to life satisfaction.

## 2 Materials and methods

### 2.1 Study design and participants

This study adopted a cross-sectional quantitative research design to examine how exercise participation influences psychological wellbeing, social connectedness, and life satisfaction among older adults. Participants were recruited from community fitness centers, senior welfare facilities, and public health programs across four metropolitan areas in South Korea.

A total of 450 paper-based questionnaires were distributed, and 412 valid responses were obtained after excluding incomplete or inconsistent entries. Participants were required to be 60 years of age or older, physically independent, and to engage in some form of regular exercise at least once a week. The sample consisted of 187 males (45.4%) and 225 females (54.6%), with an average age of 68.2 years (SD = 5.9).

### 2.2 Measures

All variables were measured using validated scales that have been widely used in gerontology and health psychology. Each scale was translated and back-translated into Korean to ensure conceptual and linguistic equivalence.

- Exercise participation was measured using three items derived from the International Physical Activity Questionnaire – Short Form (IPAQ-SF), assessing (a) the frequency of moderate-to-vigorous physical activity, (b) the average duration of such activity per session, and (c) perceived intensity. These three components were modeled as a single latent construct rather than averaged, and no additional IPAQ scoring procedures (e.g., MET-minutes) were applied. This approach aligns with prior studies that operationalize exercise engagement as a multidimensional behavioral indicator rather than an energy expenditure estimate.
- Psychological wellbeing was measured using the six-item short form of Ryff’s Psychological Wellbeing Scale, covering self-acceptance, purpose in life, and personal growth (9).
- Social connectedness was measured using the Social Connectedness Scale–Revised, assessing perceived belonging and interpersonal closeness (10).
- Life satisfaction was measured using the Satisfaction with Life Scale (SWLS) (11).

All items used a five-point Likert scale ranging from 1 (strongly disagree) to 5 (strongly agree). Cronbach’s alpha coefficients ranged from 0.86 to 0.91 for all constructs, indicating high internal reliability.

### 2.3 Procedure and ethical considerations

The study received exemption from full institutional review board (IRB) review because it involved minimal risk and used anonymized survey responses with no identifiable personal information. All data were collected without recording names or contact information, and questionnaires were stored and analyzed in fully de-identified form. Participants were informed of the study purpose, confidentiality procedures, and their voluntary rights before providing written informed consent. The exemption was granted by the Ethics Committee of Zhejiang University, in accordance with national and institutional guidelines for research involving human subjects.

### 2.4 Data analysis

Data analysis was conducted using SPSS 27.0 and AMOS 28.0. Descriptive statistics were calculated to examine sample characteristics. Measurement validity and reliability were tested through confirmatory factor analysis (CFA), evaluating factor loadings, composite reliability (CR), and average variance extracted (AVE).

Structural equation modeling (SEM) was then employed to examine the hypothesized relationships among variables. Model fit was evaluated using several indices, including chi-square (*χ*^2^/df), comparative fit index (CFI), Tucker–Lewis index (TLI), root mean square error of approximation (RMSEA), and standardized root mean square residual (SRMR).

Indirect effects were tested through bootstrapping with 5,000 resamples and 95% confidence intervals to determine the mediation effects of psychological wellbeing and social connectedness.

All analyses were performed according to the procedures described above. The main results are presented below.

## 3 Results

### 3.1 Measurement model

The confirmatory factor analysis showed an excellent model fit (*χ*^2^/df = 2.11, CFI = 0.957, TLI = 0.946, RMSEA = 0.052, SRMR = 0.041).

All standardized factor loadings were above 0.70 and statistically significant (*p* < 0.001).

Composite reliability values ranged from 0.86 to 0.91, and average variance extracted (AVE) values ranged from 0.61 to 0.73, supporting good convergent validity.

Discriminant validity was confirmed as the square root of each construct’s AVE exceeded inter-construct correlations. Reliability and convergent validity met recommended thresholds (see Table 1).

**Table 1:** Reliability and validity indices for the measurement model.

Construct	Cronbach’s *α*	CR	AVE
Exercise participation	0.88	0.89	0.68
Psychological wellbeing	0.90	0.91	0.71
Social connectedness	0.87	0.88	0.63
Life satisfaction	0.89	0.90	0.72

### 3.2 Descriptive statistics and correlations

Descriptive statistics indicated moderate to high levels of exercise participation and wellbeing among participants.

Exercise participation was positively correlated with psychological wellbeing (*r* = 0.52, *p* < 0.001) and social connectedness (*r* = 0.47, *p* < 0.001).

Both mediators showed significant positive correlations with life satisfaction, providing preliminary support for the hypothesized model. Descriptive statistics and intercorrelations among study variables are shown in Table 2.

**Table 2:** Descriptive statistics and correlations among variables (*N* = 412).

Variable	Mean	SD	1	2	3	4
Exercise participation	3.62	0.81	—			
Psychological wellbeing	3.85	0.74	0.52***	—		
Social connectedness	3.78	0.77	0.47***	0.56***	—	
Life satisfaction	3.90	0.72	0.45***	0.63***	0.60***	—

***p < 0.001.

### 3.3 Structural model and hypothesis testing

The proposed structural model demonstrated a good overall fit to the data (*χ*^2^/df = 2.24, CFI = 0.954, TLI = 0.943, RMSEA = 0.055, SRMR = 0.046).

All hypothesized paths were statistically significant and in the predicted direction:

- Exercise participation → Psychological wellbeing (*β* = 0.52, *p* < 0.001)
- Exercise participation → Social connectedness (*β* = 0.47, *p* < 0.001)
- Psychological wellbeing → Life satisfaction (*β* = 0.39, *p* < 0.001)
- Social connectedness → Life satisfaction (*β* = 0.35, *p* < 0.001)

These findings confirm that exercise participation influences life satisfaction both directly and indirectly through the two mediators, supporting the dual-pathway model. The standardized path coefficients and hypothesis testing results are summarized in Table 3. All hypothesized paths were statistically significant and in the predicted direction, supporting the dual-pathway model.

**Table 3:** Structural model results.

Hypothesis	Path	*β*	*t*-value	Result
H1	Exercise participation → Psychological wellbeing	0.52***	10.84	Supported
H2	Exercise participation → Social connectedness	0.47***	9.75	Supported
H3	Psychological wellbeing → Life satisfaction	0.39***	7.88	Supported
H4	Social connectedness → Life satisfaction	0.35***	7.21	Supported
H5	Indirect effect (Mediation)	0.37***	Bootstrapped CI [0.26, 0.49]	Supported

***p < 0.001.

### 3.4 Mediation analysis

Bootstrapping results (5,000 resamples) confirmed significant indirect effects of exercise participation on life satisfaction:

- Through psychological wellbeing: *β* = 0.20, *p* < 0.001
- Through social connectedness: *β* = 0.17, *p* < 0.001

The total indirect effect [*β* = 0.37, 95% CI (0.26, 0.49)] accounted for approximately 43% of the total effect of exercise participation on life satisfaction. These findings highlight the psychosocial mechanisms through which exercise fosters active aging.

## 4 Discussion

This study contributes to the discourse on active aging by reframing exercise participation not simply as a health behavior, but as a psychosocial ecosystem—an environment in which both psychological growth and social cohesion are nurtured (12). While prior research has documented the physical and emotional benefits of exercise, many studies have tended to treat psychological and social aspects as separate, additive components (13). Our findings call this separation into question, showing that wellbeing and connectedness function as interdependent processes that jointly shape life satisfaction in later life (14). This interpretation resonates with eudaimonic wellbeing theory, which emphasizes meaning, purpose, and personal development beyond momentary pleasure (4).

Furthermore, guided by the social ecology of aging perspective, this study emphasizes that wellbeing arises through a dynamic interaction between individuals and their environments, rather than from isolated behaviors (15). In this view, the setting and context of physical activity—such as peer interactions, shared goals, and group belonging—are as important as the activity itself in promoting holistic health (16).

### 4.1 Theoretical contributions

This research advances theoretical understanding in aging and health by introducing the concept of exercise as a psychosocial ecosystem (17). This conceptualization marks a departure from conventional models that treat physical activity as an isolated behavior or a simple means to an end.

Compared to Self-Determination Theory, which centers on individual motivation and autonomy, our framework situates wellbeing within a more relational context, where psychological and social factors are deeply interconnected (18). Social connectedness, in this model, is not a secondary benefit but a foundational element of mental resilience (19).

Likewise, while Activity Theory emphasizes continued engagement and role replacement in later life, it often treats psychological and social wellbeing as parallel outcomes (16, 20). In contrast, our dual-pathway model shows that emotional and social health reinforce each other and jointly mediate the effect of exercise on life satisfaction.

The Successful Aging Framework (24), with its focus on functional health and cognitive capacity, also leaves out important psychosocial dimensions (19, 21). Our findings suggest that older adults derive wellbeing not only from physical functioning but also from the fulfillment of deeper psychological needs—such as meaning and belonging—which are central to eudaimonic wellbeing (14).

Overall, the “psychosocial ecosystem” model offers a more integrative and socially embedded view of active aging. It brings together behavioral, emotional, and relational elements, highlighting the complex ways in which physical activity serves as a site of both personal growth and social integration.

### 4.2 Practical and policy implications

Viewing exercise through this psychosocial lens has several implications for practice and policy. Programs that aim to promote physical activity among older adults should move beyond individual fitness goals and consider how to foster shared experiences, emotional support, and social inclusion (22). Initiatives that incorporate group-based exercise, peer mentoring, and collaborative goal setting are likely to be more effective and sustainable. In practical terms, community-based formats such as group walking clubs, low-impact aerobics classes, tai chi groups, and senior community dance programs may be especially effective because they naturally facilitate peer interaction, shared goals, and social bonding. These formats support not only physical engagement but also eudaimonic wellbeing by fostering autonomy, purpose, and interpersonal connection (23).

From a policy standpoint, this model can inform the design of age-friendly environments that integrate health, social, and recreational services. Building interconnected systems that promote both psychological empowerment and social connection aligns with the WHO’s framework for healthy aging, which emphasizes participation, inclusion, and equity (4, 23).

### 4.3 Limitations and future directions

Although the structural equation model demonstrated significant associations among the study variables, the cross-sectional design does not allow for establishing temporal order or causal direction. Therefore, the mediation pathways identified here should be interpreted as associative rather than causal, even though they are consistent with the proposed theoretical model. Several limitations should be acknowledged. The cross-sectional design limits causal inference, and future research should employ longitudinal or experimental approaches to better examine temporal relationships between exercise, wellbeing, and social connectedness. In addition, while this study focused on offline community settings, the growing relevance of digital platforms—especially in post-pandemic contexts—warrants exploration. Future studies could investigate how virtual exercise communities and online social interactions influence eudaimonic wellbeing and social vitality in aging populations. Additionally, because participants were recruited from community fitness centers, senior welfare facilities, and wellness programs, the sample may overrepresent older adults who are already more physically active or socially engaged than the general population. This recruitment context should be considered when interpreting the findings, as it may limit the generalizability of the results to less active or socially isolated older adults.

## 5 Conclusion

This study demonstrates that exercise participation plays a vital role in promoting active aging, not merely as a physical health behavior but as a psychosocial ecosystem that integrates mental and social wellbeing. The findings reveal that physical activity contributes to life satisfaction through interconnected emotional and relational mechanisms, emphasizing that health in later life is both psychological and social.

By reframing exercise within a eudaimonic and social-ecological perspective, this research extends the conceptual boundaries of active aging. It highlights that the meaning of exercise lies not only in improving fitness but also in fulfilling the intrinsic human needs for purpose, relatedness, and belonging.

These insights contribute to the growing recognition that successful aging must be supported by both individual agency and relational environments. Rather than isolating physical health from emotional or social wellbeing, public health strategies should embrace the interconnectedness of these domains.

Practically, this framework calls for the design of community-based exercise initiatives that foster both emotional resilience and social inclusion. Such integrative programs can serve as public health catalysts, supporting longer, healthier, and more connected lives.

Future research should employ longitudinal and cross-cultural designs to examine how evolving social environments—including digital communities—can sustain eudaimonic wellbeing and social vitality across diverse aging populations. These findings reinforce the eudaimonic view that purposeful engagement, rather than mere activity, sustains wellbeing in later life.

## Data Availability

The datasets generated for this study are not publicly available due to ethical and legal considerations. Requests to access the data may be considered by the corresponding authors on a case-by-case basis, and the authors reserve the right to decline data sharing where appropriate.
