# Correction: From behavior to belonging: reframing exercise participation as a psychosocial pathway to active aging

**DOI:** 10.3389/fpubh.2026.1869576

**Published:** 2026-05-15

**Authors:** Soo-Jin Choi, Fang Zheng

**Affiliations:** Zhejiang University, Hangzhou, China

**Keywords:** active aging, eudaimonic wellbeing, exercise participation, psychosocial health, public health policy, social ecology, social connectedness

The reference for Reference 10 was erroneously written as 10. Kim, Y, and Cho, H. Active leisure, life satisfaction, and health promotion among retirees. Leisure Stud. (2020) 39:829–845. doi: 10.1080/02614367.2020.1740695 It should be 10. Nimrod G. Retirees' leisure: activities, benefits, and their contribution to life satisfaction. Leisure Stud. (2007) 26:65–80. doi: 10.1080/02614360500333937

The reference for Reference 12 was erroneously written as 12. Loh V, Fung HH. Social motivation and active engagement across the lifespan. Psychol Aging. (2020) 35:389–403. doi: 10.1037/pag0000451. It should be 12. Kobayashi LC, Steptoe A. Social isolation, loneliness, and health behaviors at older ages: longitudinal cohort study. *Ann Behav Med*. (2018) 52:582–93. doi: 10.1093/abm/kax033.

The reference for Reference 15 was erroneously written as 15. Ribeiro F, Oliveira N, Sousa N, et al. The impact of community-based exercise on older adults' wellbeing. Healthcare (Basel). (2022) 10:721. doi: 10.3390/healthcare10040721. It should be replaced with reference 4. Ryff CD. Happiness is everything, or is it? Explorations on the meaning of psychological well-being. *J Pers Soc Psychol*. (1989) 57:1069. doi: 10.1037/0022-3514.57.6.1069.

The reference for Reference 17 was erroneously written as 17. Silverstein, M, and Bengtson, VL. Does intergenerational social support influence the psychological well-being of older parents? *Gerontologist*. (1994) 34:34–43. doi: 10.1093/geront/34.1.3. It should be 17. Silverstein M, Bengtson VL. Does intergenerational social support influence the psychological well-being of older parents? *Gerontologist* (1994) 34:34–43. doi: 10.1016/0277-9536(94)90427-8.

The reference for Reference 20 was erroneously written as 20. Tse, ACY, Wong, TWL, and Lee, PH. The role of exercise self-efficacy in promoting active aging. *J Aging Phys Act*. (2020) 28:345–357. doi: 10.1123/japa.2018-0417. It should be 20. Southwick SM, Bonanno GA, Masten AS, Panter-Brick C, Yehuda R. Resilience definitions, theory, and challenges: interdisciplinary perspectives. *Eur J Psychotraumatol*. (2014) 5:25338. doi: 10.3402/ejpt.v5.25338.

The original version of this article has been updated.

